# Pigment Epithelium-Derived Factor (PEDF) Interacts with Transportin SR2, and Active Nuclear Import Is Facilitated by a Novel Nuclear Localization Motif

**DOI:** 10.1371/journal.pone.0026234

**Published:** 2011-10-18

**Authors:** Sergio Anguissola, William J. McCormack, Michelle A. Morrin, Wayne J. Higgins, Denise M. Fox, D. Margaret Worrall

**Affiliations:** UCD School of Biomolecular and Biomedical Science, Conway Institute, University College Dublin, Belfield, Dublin, Ireland; University of South Florida College of Medicine, United States of America

## Abstract

PEDF (Pigment epithelium-derived factor) is a non-inhibitory member of the serpin gene family (serpinF1) that displays neurotrophic and anti-angiogenic properties. PEDF contains a secretion signal sequence, but although originally regarded as a secreted extracellular protein, endogenous PEDF is found in the cytoplasm and nucleus of several mammalian cell types. In this study we employed a yeast two-hybrid interaction trap screen to identify transportin-SR2, a member of the importin-β family of nuclear transport karyopherins, as a putative PEDF binding partner. The interaction was supported *in vitro* by GST-pulldown and co-immunoprecipitation. Following transfection of HEK293 cells with GFP-tagged PEDF the protein was predominantly localised to the nucleus, suggesting that active import of PEDF occurs. A motif (YxxYRVRS) shared by PEDF and the unrelated transportin-SR2 substrate, RNA binding motif protein 4b, was identified and we investigated its potential as a nuclear localization signal (NLS) sequence. Site-directed mutagenesis of this helix A motif in PEDF resulted in a GFP-tagged mutant protein being excluded from the nucleus, and mutation of two arginine residues (R67, R69) was sufficient to abolish nuclear import and PEDF interaction with transportin-SR2. These results suggest a novel NLS and mechanism for serpinF1 nuclear import, which may be critical for anti-angiogenic and neurotrophic function.

## Introduction

Pigment epithelium-derived factor (PEDF) is a 50 kDa glycoprotein and a non-inhibitory member of the serine protease inhibitor (serpin) superfamily, originally identified as a neurotrophic factor secreted by retinal pigment epithelial (RPE) cells [1], [2]. In addition to a neuroprotective role, PEDF is also a potent inhibitor of angiogenesis [3] and can inhibit vascular endothelial growth factor (VEGF) induced vasopermeability in the eye [4]. A PEDF-null mouse displayed increased vasculature in the prostate and pancreas [5]. More recently adipocyte released PEDF has ben associated with insulin resisatnce and inflammatory signalling in muscle and fat cells [6].

Specific targets that mediate the mechanism of action of extracellular PEDF remain unclear. A lipase-linked membrane receptor (PEDF-R) has been identified [7], and a yeast-two-hybrid screen has revealed the non-integrin laminin receptor as a potential target [8]. Peptides derived from PEDF have been elucidated in terms of structure- function relationships [9]. A region of the molecule spanning amino acids 44–121 contains 2 biologically active peptides, a 34-mer peptide with anti-angiogenic activity, and a 44-mer peptide promoting neuronal differentiation [10]. Mapping of sites for PEDF binding to extracellular matrix components has revealed a positively charged region for heparin binding and a cluster of acidic amino acids responsible for collagen binding [11].

PEDF is generally regarded as a secreted protein, but several immunohistochemical studies have reported intracellular protein detection including strong nuclear staining [12]–[14]. Using subcellular fractionation, Tombran-Tink et al [15] showed that endogenous PEDF was present in the cytoplasmic and nuclear fractions of retinal pigment epithelial cells (RPE), Y-79 retinoblastoma cells, NA neuroblastoma cells and hepatocarcinoma HepG2 cells. In a separate study [16], expression of PEDF was seen in the nuclei of hepatocytes, but was mainly cytoplasmic in hepatocellular carcinoma cells raising the possibility that PEDF localization may have functional significance for disease.

In this study we initially carried out a yeast-2-hybrid screen to identify potential novel interactants using a bait of 81 amino acids (amino acids 41–121) containing the minimum known structural determinants for biological activity. A putative interaction with transportin-SR2, a member of the importin-beta family was found, and alignment with an unrelated transportin substrate RBM-4b revealed a shared motif which we hypothesise to be a novel NLS sequence. Following mutagenesis of this helix A motif in GFP-tagged PEDF, we find complete exclusion from the nucleus, with two basic residues (R67 and R69) being critical for nuclear import and transportin-SR2 interaction.

## Results

### Yeast-2-hybrid screening with PEDF^41–121^ identifies TRN-SR2 as a potential interaction candidate

**Figure 1:**
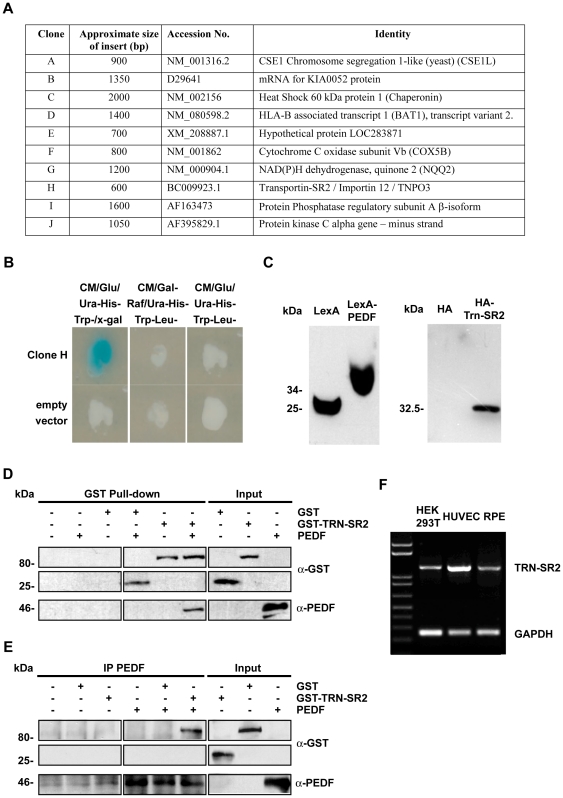
PEDF interaction with Transportin-SR2 identified by Yeast two Hybrid screening and confirmed by *in vitro* interaction of recombinant proteins. pEG202PEDF^41–121^ was transformed into EGY48 yeast strain, subsequently transformed with a foetal human brain library. Blue-white selection in presence of X-Gal identified possible interaction candidates. A. List of unique Yeast two Hybrid clones expressing PEDF^41–121^ interaction candidates. B. Positive interactions identified by the screening. Clone H expressing LexA- PEDF^41–121^ and HA-TRN-SR2, and EGY48 yeast transformed with pEG202 empty vector were grown on different selection media as indicated. C. Verification of bait and target fusion protein expression. pEG202PEDF^41–121^ and pJG4-5-TRN-SR2^827–923^ recovered from clone H were transformed into yeast; As controls, empty vectors pJG4-5 and pEG202 were also transformed into yeast. Yeast lysates were subjected to immunoblotting using a polyclonal anti-LexA and a polyclonal anti-HA antibody. D. Verification of PEDF and TRN-SR2 interaction by GST-pull down. GST, GST-TRN-SR2 full length and PEDF were cloned and purified according to the methods. GST or GST-TRN-SR2 were incubated with glutathione-agarose for 1 hr followed by addition of recombinant PEDF for 4 hr at 4°C. Samples were subjected to immunoblotting using a monoclonal GST, a monoclonal PEDF and monoclonal Transportin-SR2 antibody. E. Verification of PEDF and TRN-SR2 interaction by co-immunoprecipitation. Recombinant PEDF was incubated with GST or GST-TRN-SR2 for 4 h and monoclonal PEDF antibody pre-absorbed on Protein-A agarose for 1 hr at 4°C Samples were subjected to immunoblotting using a monoclonal anti-GST, a monoclonal anti-PEDF and monoclonal anti-Transportin-SR2. F. RT-PCR detection of TRN-SR2 expression in HEK293 cells used for transfections in this study, and in retinal pigment epithelial cells and HUVEC cells known to contain nuclear PEDF.

The yeast-2-hybrid system used for this study consisted of a LexA DNA binding domain bait fusion and the activation domain (B42 from VP16) target fusion library and was identical to that previously reported for maspin interactant identification [17]. A human fetal brain cDNA library in pJG-45 was screened with an 81 amino acid region of PEDF; this region is thought to be the active domain of PEDF responsible for its anti-cancer and anti-angiogenic functions [10], [11]. Prior to screening the pEG202-PEDF^41–121^ bait was shown to be transcriptionally inert for both the LEU2 and LacZ reporter genes (data not shown). As a result of the library screen 10 unique clones which activated expression of both LEU2 and LacZ reporter genes were isolated and sequenced (Fig. 1A). Of particular interest was transportin-SR2/ Transportin-3/ TNPO3, a member of the importin-beta superfamily of proteins that act as karyopherins of serine/arginine (SR)-rich mRNA splice proteins and other non SR-proteins including HIV-integrase [18], [19]. The sequenced transportin-SR2 (*TRN-SR2*) clone revealed that the insert comprised 251 bases of the 3′untranslated region and 291 bases (1892–2183) of the coding region (GenBank Accession Number NM_012470) which correspond to the C-terminal 96 amino acids of TRN-SR2. The identified C-terminal region (amino acids 827 to 923) was in-frame and constitutes part of the domain known to bind cargo proteins [20]. As an example of the yeast-two-hybrid interaction, cultures of clone H expressing pEG202-PEDF^41–121^ and pJG-45-TRN-SR2^827–923^ compared to a clone expressing pEG202-PEDF^41–121^ and pJG-45 on different selection media are shown (Fig. 1B). pEG202-PEDF^41–121^ and pJG-45-TRN-SR2^827923^ were transformed into yeast EGY48 and expression of both bait and target fusion proteins was assessed by immunoblotting using LexA and HA antibodies (Fig. 1C, D), revealing the correct predicted protein size in each case.

To further characterize the interaction between PEDF and TRN-SR2 the cDNA encoding for full length proteins were cloned into expression vectors as described in the methods section; GST-pull down and co-immunoprecipitation experiments were performed followed by immunoblotting, revealing a specific interaction between PEDF and TRN-SR2 and excluding non specific cross-interaction with either GST, glutathione agarose or the PEDF antibody (Fig. 1E).

Transportin-SR2 and the related transportin-SR [21] are alternative splice products of the same gene (*TNP03*) but the expressed form in most tissues and cell lines appears to be TRN-SR2 [22]. Gene expression databases show that transportin-SR2 or transportin-3 (TNP03) is a widely distributed protein with expression detected in 130 human tissues and organs, and expression found at all stages of human development. (http://bgee.unil.ch/bgee/bgee?page=gene&action=expression&gene_id=ENSG00000064419)

Sources include retina, liver, brain adipose tissue and other tissues known to express PEDF also, and in the case of retina and liver, nuclear localisation of PEDF has been reported [14], [15]. Using RT-PCR with primers specific to *TRN-SR2*, we also verified TRN-SR2 expression in the HEK293T cell line used in this study for localisation experiments, in HUVECs (Human Umbilical Vein Endothelial Cells) and in retinal pigment epithelial cells from which PEDF was originally isolated (Figure 1F). However, endogenous levels of TRN-SR2 protein proved difficult to detect using anti-TNP03 antibody.

### Putative Nuclear Localization Sequences

**Figure 2:**
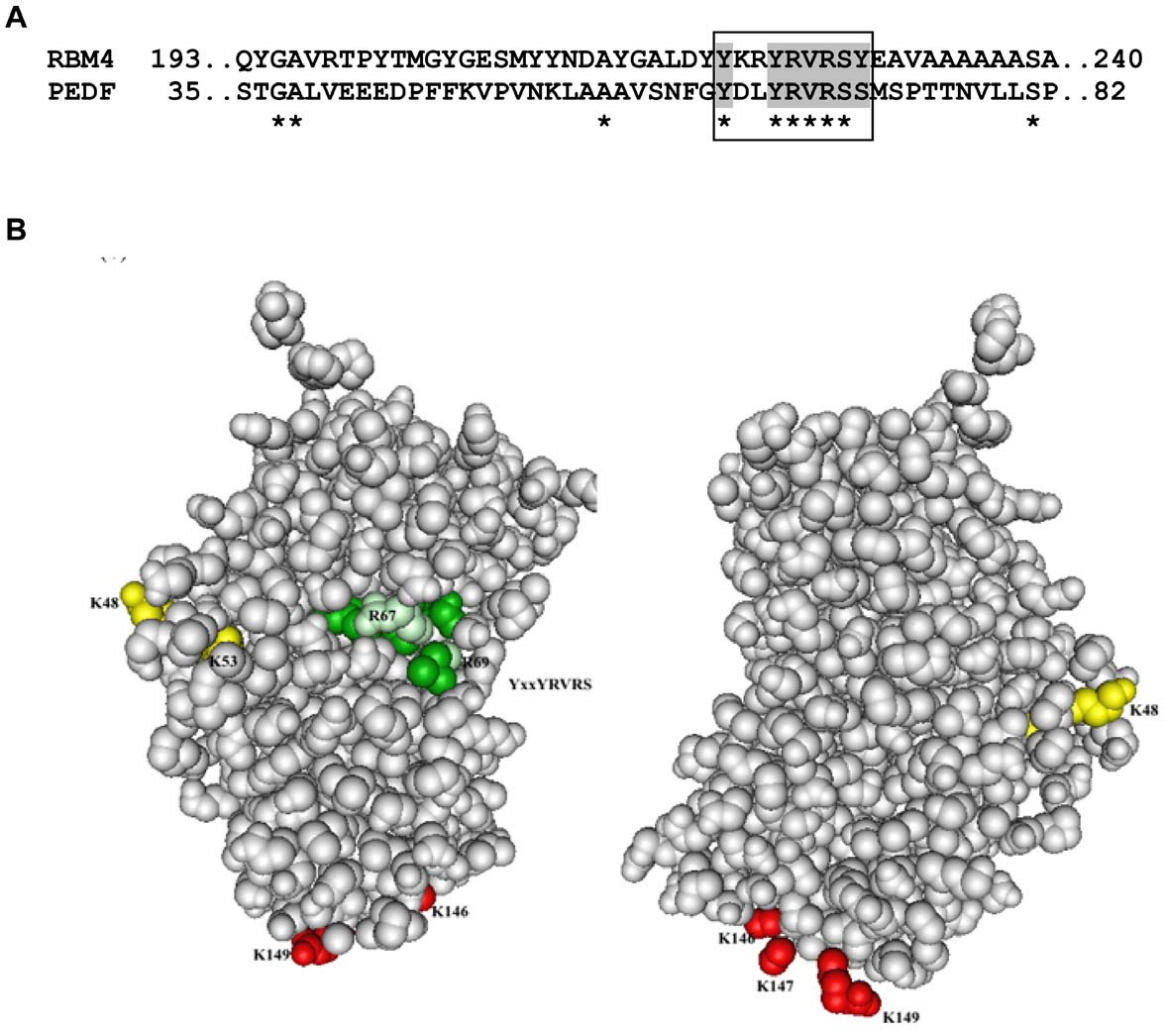
Identification of putative PEDF nuclear import motifs. A. Alignment of PEDF with the known transportin-SR2 substrate cargo RNA binding protein, RBM4b (Genbank Acc. AAH04951). The RBM4b C-terminal domain (amino acids 196–364) interacts with TRN-SR2 [20]. B. Potential NLS residues highlighted in the crystal structure of PEDF [24]. The novel YxxYRVRS motif is found in helix A (green), with possible linked bipartite lysine residues in yellow. The basic residues important for heparin binding are shown in red.

TRN-SR2 is known to facilitate import of phosphorylated SR-rich proteins and non- SR-rich proteins involved in RNA processing [20], [23]. We have performed sequence comparisons between PEDF and other TRN-SR2 substrates to identify possible similarities. Fig. 2A shows an alignment between the N-terminal region of PEDF and the known TRN-SR2 binding domain of RNA-binding motif protein 4 (RBM4b, also annotated as RNA binding motif protein 30), a non-SR substrate of TRN-SR2 [20]. Although these proteins are unrelated structurally and functionally, this alignment has surprisingly revealed a common motif (YxxYRVRS) and we hypothesised that this might be a novel nuclear localization signal sequence. In the PEDF sequence this motif comprises residues 63 to 70, found in helix A and highlighted in the crystal structure of PEDF [24] (Fig. 2B). Classical nuclear localization signal sequences generally appear either as a single-stretch or as two small clusters of basic residues separated by approximately 12 amino acid residues, with the consensus sequences for these monopartite and bipartite basic NLS's being (K/R)4–6 and (K/R)2 X10–12(K/R)3 respectively. On examination of upstream and downstream sequences to the YxxYRVRS motif, two positively charged lysine residues are observed 12 and 16 residues N-terminal to Arg67 of the YxxYRVRS motif. Residues K48 and K53 on the PEDF structure (Fig. 2B) together with Arg67 and Arg69 within the YxxYRVRS motif, i.e. (K48K53)X12(R67R69), could constitute a non-classical bipartite NLS. An alternative potential NLS has been suggested by others as the positively charged cluster of basic amino acids known to be critical for heparin binding [15] (highlighted at the base of the molecule in Fig. 2B). These residues could possibly represent a monopartite NLS.

### Active nuclear import of PEDF is mediated by the YXXYRVRS motif

**Figure 3:**
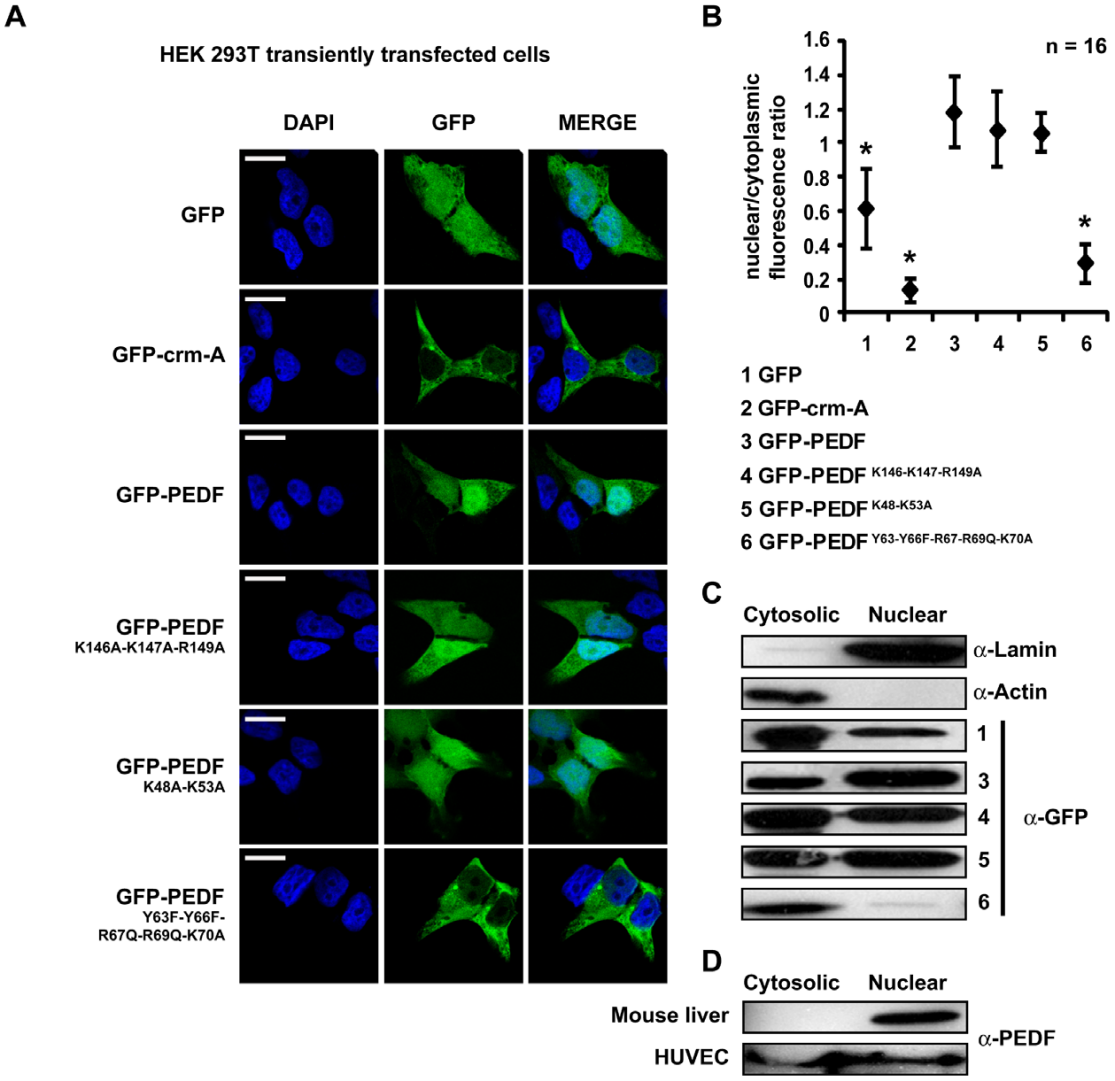
Mutation of the YxxYRVRS motif blocks nuclear import of PEDF. HEK293T cells were transiently transfected with cDNA coding for GFP, GFP-PEDF wt and the following mutants: GFP-PEDF^K146A-K147A-R149A^; GFP-PEDF^K48A-K53A^; GFP-PEDF^Y63F-Y66F-R67Q-R69Q-K70A^. A. Transiently transfected cells were fixed, stained with DAPI and analyzed by confocal microscopy. As negative control for nuclear import, cells were transiently transfected with GFP-crm-A. B. Nuclear localization of GFP, GFP-PEDF and mutants was assessed in transiently transfected cells as a ratio of nuclear to cytoplasmic fluorescence using the Zeiss LSM 510 software. Data are means +/− SD from n = 16 fluorescent cells analyzed. * p<0.05 difference from GFP-PEDF transfected cells. Scale bar = 20 µm. C. Cells transiently transfected with GFP-PEDF and mutants were subjected to subcellular fractionation. The cytosolic and nuclear fractions were subjected to immunoblotting using a polyclonal GFP antibody. A polyclonal anti-lamin and a monoclonal anti-actin antibody were used as nuclear and cytoplasmic controls respectively. D. Cytosolic and nuclear fractions from HUVEC cells and mouse liver tissue, subjected to immunoblotting with anti-PEDF polyclonal antibody to detect nuclear localisation of endogenous PEDF.

Several studies have shown that endogenous PEDF is present in the nucleus of different cell types [12]–[14]. Native PEDF prior to glycosylation is 45 kDa and therefore is close to the limit for diffusion into the nucleus [25]. To investigate whether PEDF actively translocates to the nucleus and to elucidate the functional NLS, a GFP fusion protein was designed with a molecular weight of 75 kDa which is too large for passive nuclear diffusion. This approach has been previously used to show active nuclear import of several B-clade serpins [26]. PEDF was cloned into the pEGFP-C1 vector and transiently transfected into HEK293T cells. Cells were then fixed, stained with the nuclear dye DAPI and analyzed by confocal microscopy. While GFP fluorescence homogeneously distributes in the cytosol and nucleus, the GFP-PEDF signal appears to be predominantly accumulating in the nucleus (Fig. 3A). A control transfection with GFP fused to CrmA, a viral serpin with similar size to PEDF, was shown to be excluded from the nucleus (Fig. 3A) indicating that the GFP moiety is not capable of facilitating serpin nuclear import. The intracellular distribution in each case was confirmed by quantitative analysis of GFP nuclear to cytoplasmic fluorescence ratio (Fig. 3 B).

To characterize the sequence involved in PEDF nuclear import we generated mutants of the heparin binding region, the YXXYRVRS motif and the upstream lysine residues for their potential as part of a functional NLS sequence.

For the YXXYRVRS motif, we initially altered five PEDF amino acids (Y63F, Y66F, R67Q, R69Q, S70A). These amino acids are not highly conserved in serpins generally (for full alignment see http://supfam.org/SUPERFAMILY/); many contain phenylalanine rather than tyrosine in positions 63 and 66 (PEDF numbering), and a number of serpins have glutamine residues in positions equivalent to PEDF Arg67 and Arg69. To investigate the possibility that the lysine residues at positions 48 and 53 may form a bipartite NLS sequence with the YxxYRVRS motif, we mutated this positively charged cluster separately (K48A, K53A). The basic residues involved in heparin binding (region146–149) suggested as a potential NLS [15] were also changed to alanines as a single mutant construct (K146A, K147A, R149A). The individual mutants cloned into pEGFP-C1 were transfected into HEK293T cells. Cells were fixed, stained with DAPI and subjected to confocal microscopy analysis as above. Mutation of either the heparin binding/putative NLS motif or the K48 /K53 residues did not interfere with GFP-PEDF accumulation into the nucleus while mutation of our proposed motif (Y63, Y66, R67, R69, S70) completely excluded GFP-PEDF from the nucleus (Fig. 3A). The observations were confirmed by analysis of the nuclear to cytoplasmic distribution of GFP fluorescence (Fig. 3B). To further verify the intracellular localization of GFP-PEDF and mutants, transiently transfected cells were subjected to cell fractionation followed by SDS-PAGE and immunoblotting; GFP-PEDF, GFP-PEDF^K48A/K53A^ and GFP-PEDF^K146A/K147A/R149A^ showed an increased nuclear∶cytoplasmic ratio compared to GFP while GFP-PEDF^Y63F/Y66F/R67Q/R69Q/K70A^ was undetectable in the nuclear fraction (Fig. 3B).

### Arginines 67 and 69 are required for PEDF nuclear import

**Figure 4:**
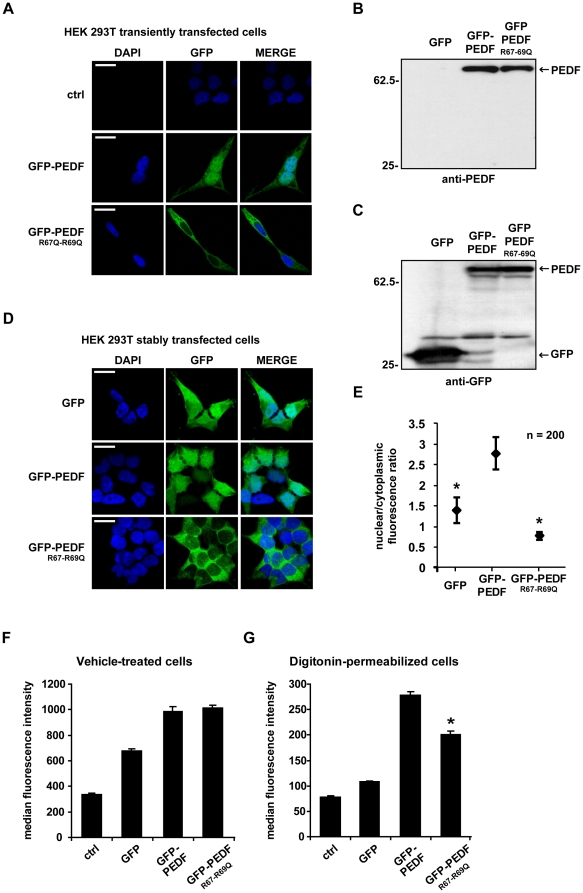
Arginine 67 and 69 are required for PEDF nuclear accumulation in transiently and stably transfected cells. HEK293T cells were transiently transfected with cDNA coding for GFP-PEDF and GFP-PEDF^R67Q-R69Q^. Transfected cells were incubated with G418 (500 µg/ml) for 14 days and stable transfected fluorescent cells were enriched by fluorescence-activated cell sorting (FACS). A. Transiently transfected cells were fixed, stained with DAPI and analysed by confocal microscopy. Scale bar = 20 µm. B, C. Stably transfected cells were lysed and subjected to immunoblotting using a polyclonal GFP and a monoclonal PEDF antibody. D, E. Stably transfected cells were fixed, stained with DAPI and analyzed by confocal microscopy. 40× images were collected and nuclear localisation of GFP, GFP-PEDF and GFP- PEDF^R67Q-R69Q^ was expressed as ratio of nuclear to cytoplasmic fluorescence. Data expressed as mean +/− SD from n = 200 cells analyzed. * p<0.05 difference from GFP-PEDF transfected cells. F, G. Control and stably transfected cells were incubated with vehicle (PBS) or permeabilized with digitonin (2 µg/ml) for 10 min at 4°C. Fluorescent emission was acquired using flow cytometry. Data are means +/− SD from n = 3 separate experiments. * p<0.05 difference from GFP-PEDF transfected cells. Scale bar = 20 µm.

Given that basic residues are most critical in nuclear localization motifs, we investigated the importance of the 2 arginine residues within the identified NLS. A pEGFP-PEDF^R67Q/R69Q^ mutant was generated and transiently transfected into HEK293T cells; confocal microscopy analysis revealed that the PEDF mutant is excluded from the nucleus (Fig. 4A). To further characterize this observation, HEK293T cells were stably transfected with pEGFP, pEGFP- PEDF, pEGFP-PEDF^R67Q/R69Q^ and the fluorescent cells were enriched by cell sorting. Cell lysates were subjected to immunoblotting to verify correct protein expression in the stable cell lines (Fig. 4B, C). In non-transfected or transfected HEK293 cells no endogenous PEDF was detected; overexposure of the anti-PEDF immunoblot shown in Figure 4B gave no signal at approximately 45 kDa which would be expected for endogenous PEDF expression. Confocal microscopy analysis showed comparable results to transiently transfected cells (Fig. 4D), and analysis of the nuclear to cytoplasmic fluorescence ratio confirmed the previous results. PEDF shows a high degree of co-localization with the nuclear staining DAPI, and a higher nuclear vs cytoplasmic ratio than the mutant PEDF ^R67Q/R69Q^ (Fig. 4E). To further validate our finding a different approach was used whereby stably transfected HEK293T cells were subjected to digitonin permeabilization in order to release the cytoplasmic content from the cells but retain the nucleus and other intracellular organelles. Non-permeabilized cells (Fig. 4F) and digitonin-permeabilized cells (Fig. 4G) were analyzed by flow cytometry and the total fluorescence was recorded. In non-permeabilized cells GFP-PEDF and GFP-PEDF^R67Q/R69Q^ expressing cells show very similar GFP fluorescence intensity, but when cells are permeabilized with digitonin the residual nuclear fluorescence is significantly lower in GFP- PEDF^R67Q/R69Q^ expressing cells (Fig. 4G), indicating that GFP-PEDF^R67Q/R69Q^ was predominantly cytoplasmic. A control experiment performed by time-lapse confocal microscopy confirmed complete release of intracellular GFP from stably transfected GFP expressing HEK 293T cells (data not shown).

### Arginines 67 and 69 are required for PEDF interaction with TRN-SR2

**Figure 5:**
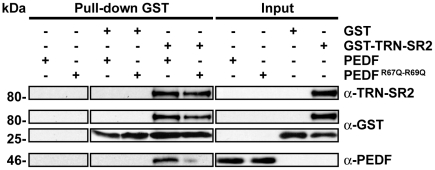
Arginine 67 and 69 are required for PEDF interaction with Transportin-SR2. GST and GST-TRN-SR2 were purified on glutathione-agarose from BL21 *E.coli*. Human recombinant PEDF and PEDF^R67Q-R69Q^ were purified on heparin-agarose from medium of transiently transfected HEK293T cells. GST or GST-TRN-SR2 were incubated with glutathione-agarose for 1 hr followed by addition of recombinant PEDF or PEDF^R67Q-R69Q^ for 4 h at 4°C. Samples were subjected to immunoblotting using a monoclonal GST, a monoclonal PEDF and a monoclonal Transportin-SR2 antibody.

Mutagenesis of Arg 67 and Arg 69 abolishes PEDF nuclear import and this phenomenon could be caused by impaired interaction with nuclear importins. To verify requirement of these residues for the interaction between PEDF and TRN-SR2 site directed mutagenesis, as above, was performed with the mammalian expression vector, pCMV5.0PEDF, and recombinant proteins were expressed and purified as described in the methods. GST pull down experiments were performed followed by immunoblotting, revealing that the specific interaction between PEDF and TRN-SR2 was impaired when PEDF^R67Q/R69Q^ was incubated with GST-TRN-SR2 (Fig. 5). Non specific interaction with either GST, glutathione agarose or the PEDF antibody was tested and resulted negative (Fig. 5).

**Figure 6:**
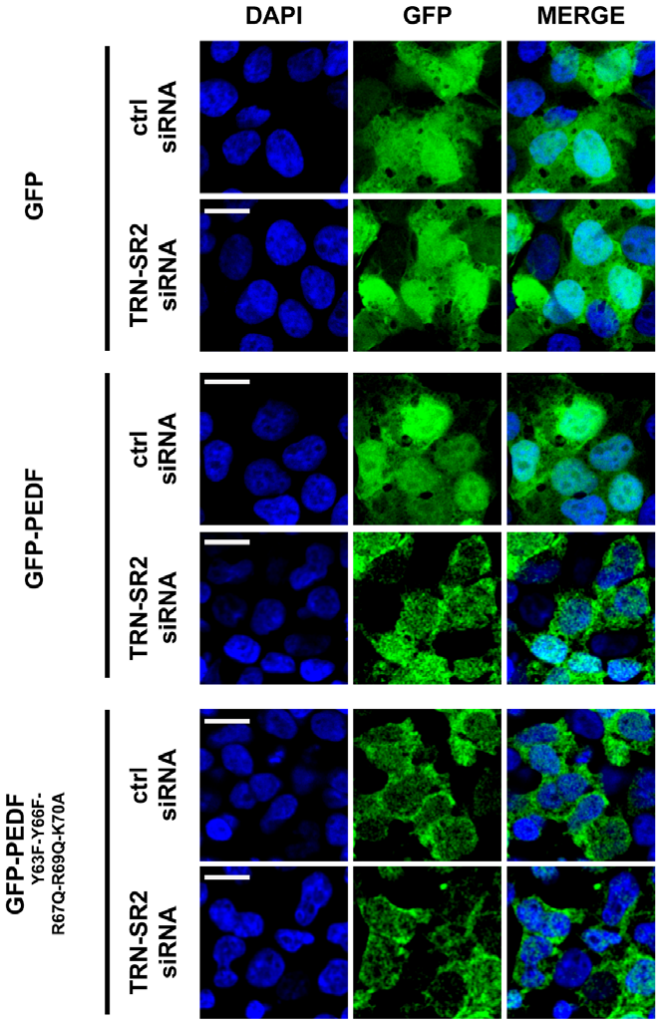
Transportin SR2 knock down inhibits nuclear accumulation of GFP-PEDF. HEK293T cells stably transfected with cDNA coding for GFP, GFP-PEDF and GFP-PEDF^R67Q-R69Q^ were transfected with a scrambled siRNA (ctrl siRNA) or with a Transportin SR2 siRNA (TRN-SR2 siRNA). After 48 hours cells were fixed, stained with DAPI and analyzed by confocal microscopy. 63× images were collected and nuclear localisation of GFP, GFP-PEDF and GFP- PEDF^R67Q-R69Q^ was observed. Scale bar = 20 µm.

In order to verify that TRN-SR2 is required for PEDF nuclear translocation we inhibited the expression of TRN-SR2 in HEK293T cells stably transfected with GFP, GFP-PEDF and GFP- PEDF^R67Q/R69Q^. For this purpose these cell lines were transfected with synthetic oligonucleotides using nucleotide sequences that were previously shown to reduce TRN-SR2 expression and reduce HIV nuclear import [27]. PEDF cellular distribution was observed by confocal microscopy 48 hours after transfection. As shown in Fig. 6, a mismatched siRNA (ctrl siRNA) does not alter GFP cellular distribution in the 3 cell lines; TRN-SR2 siRNA does not alter nuclear accumulation of GFP and does not affect nuclear exclusion of GFP- PEDF^R67Q/R69Q^ but it significantly decreases nuclear accumulation of GFP-PEDF, supporting the hypothesis that TRN-SR2 is an important component of the protein machinery that shuttles PEDF in the nucleus.

## Discussion

Although PEDF has traditionally been regarded as a secreted extracellular protein, there is increasing evidence for a significant intracellular presence of the protein in several mammalian tissues and cells. A comprehensive study has shown both cytoplasmic and nuclear levels of PEDF in retinal pigment epithelial cells (RPE), Y-79 retinoblastoma cells, NA neuroblastoma cells and hepatocarcinoma HepG2 cells [15]. Our results indicate that transport of PEDF into the nucleus is an active process and PEDF can now be included with a growing list of human serpins displaying a nuclear or nucleocytoplasmic distribution, including antichymotrypsin, angiotensinogen and the B-clade serpins, B6, B8 and B9 and B10. Serpin B10 (bomapin) contains a classical NLS [28] but others lack any obvious signal sequence, and Bird *et al* [26] proposed that an importin-beta family member was likely to be involved in nuclear import of serpin B9. We have now revealed this to be the case for PEDF using a yeast-2-hybrid approach, a method has been previously used to identify receptor targets for PEDF [7], [8]. The interaction with the importin-b family member transportin-SR2/transportin 3 has enabled us to elucidate a functional helix A NLS for PEDF. This motif does not appear to be present in other nucleocytoplasmic serpins mentioned above. However, we suggest that it is likely to be essential for RBM4b import and for other transportin-SR2 substrates, if present. Interestingly, mutagenesis of a similar RVR sequence within the NLS of the human TAP protein can also block the observed transportin-dependant NLS function [29].

PEDF does contain a secretion signal sequence and is thought to mediate its neurotrophic and antiangiogenic effects as a secreted extracellular protein. The active nuclear uptake of PEDF found here opens up two possibilities. One is that PEDF has a dual role in cells expressing the protein whereby some product is secreted, and some is transported to the nucleus. Such a dual specificity has been proposed for other secreted serpins such as PAI-2 and MNEI. More relevant are the plasma serpins α1-antichymotrypsin and angiotensinogen, which like PEDF have a strong N-terminal secretion signal sequence, but also have a nuclear localization, with antichymotrypsin demonstrating direct binding to double stranded DNA [30]. There is some evidence that intracellular PEDF may lack the secretion signal either through posttranslational processing or via mRNA splice variants. Although a 50 kDa protein was detected in the interphotoreceptor matrix of the retina, soluble extracts of retinal pigment epithelial cells contained little of the 50 kDa species but contained an immunoreactive 36 kDa protein [12].

A second possibility is that extracellular PEDF may act on target cells by internalization and subsequent translocation to the nucleus. There are a number of precedents for such trafficking among growth factors and neurotrophic agents, including nerve growth factor, the acidic fibroblast growth factor, Schwannoma derived growth factor and midkine [31]. Cellular uptake followed by nuclear import of midkine was found to be essential for its promotion of cell survival [32]. Studies with fluorescein labelled PEDF binding to rat spinal cord motorneurones by Bilak *et al* [33], reported that label was consistently found in the nucleus following 12 hr incubation. This was assumed to be due to degradation products and not further investigated, but our findings raise the possibility that the extracellular PEDF ligand may have been translocated to the nucleus via endocytosis and an importin-beta dependant nuclear transport process. Other studies have shown that PEDF is rapidly cleared by the retina and by RPE following intravitreal injection [34], and recombinant PEDF was undetectable in the medium of microglial cultures 24 hours after addition [35]. These findings could be due to degradation but endocytosis of a receptor-PEDF complex may also provide an explanation.

The role of most serpins found in the nucleus is unknown. MENT, an avian serpin, localises in the nucleus and is associated with chromatin condensation and inhibition of nuclear papain-like cysteine proteases [36]. Specific B-clade serpins and antichymotrypsin may also inhibit nuclear proteases, but PEDF lacks the necessary conformational instability and is classified as a non-inhibitory serpin. PEDF could have a more direct gene regulatory role, and the involvement of PEDF (denoted EPC-1 in the study) in G_0_ growth arrest in fibroblasts has led to the suggestion of a direct role in the cell cycle [37].

In summary, the active nuclear transport of PEDF shown here is consistent with tissue and cellular immunohistochemical and immunoblotting studies reporting significant quantities of endogenous PEDF in the nucleus. Elucidation of a novel NLS motif that is critical for this import will facilitate further investigation into the relationship between PEDF localization and biological function.

## Materials and Methods

### Cell culture

The HEK293T cell line (ATCC, CRL-11268) was grown in Dulbecco's modified Eagle's medium (DMEM) supplemented with 10% (v/v) heat-inactivated fetal bovine serum, 2 mM glutamine, 100 U/ml penicillin and 100 µg/ml streptomycin (complete medium) in a humidified 5% CO_2_ containing atmosphere at 37°C. Cells were kept in logarithmic growth phase by routinely passaging them three times a week and were seeded 24 hr prior to treatments.

### Cloning, expression and site-directed mutagenesis

Primers used in the study are detailed in Table 1. The human PEDF open reading frame was reverse transcribed from mRNA obtained from hepatoma HepG2 cells (ATCC, HB-8065) using MMLV reverse transcriptase (Stratagene) with primers sPEDF and asPEDF and amplified with *Pfu* Turbo DNA polymerase (Stratagene). The sequence was verified as identical to PEDF Genebank accession number AF400442.1. PEDF was cloned into pEGFPC1 to generate an N-terminal fusion with enhanced green fluorescent protein (eGFP) using primers sGFP-PEDF and asPEDF. The EGFP-crmA vector was kindly donated by Prof. Phil Bird, Monash University, Victoria, Australia. For the yeast-two-hybrid bait construct the PEDF region encoding aminoacid residues 41 to 121 was amplified with the primers sPEDF119 and asPEDF365 and cloned into pEG202. For mammalian cell expression, a Kozak consensus sequence was inserted before the ORF initiation codon using the primer sPEDF^K^ and asPEDF, and the product was cloned into pCMV5.0.

**Table 1:** Primer Sequences.

Primer Name	Sequence 5′→3′
sPEDF	TTTGAATTCATGCAGGCCCTGGTGCTAC
asPEDF	TTGTCGACCAGCCTTCGTGTCCTGTGG
sPEDF^119^	ACGGAATTCATGGAGGAGGAGGATCCTTTC
asPEDF^365^	GTAGTCGACTTAGGTACCATGGATGTCTGG
BCO1	AGCCTCTTGCTGAGTGGAGATGC
BCO2	GACAAGCCGACAACCTTGATTGGAG
PEDF^K^	TTTGAATTCGCCACCATGGAGGCCCTGGTGCTACTC
sGFP-PEDF	ATGAATTCTATGAACCCTGCCAGCCCCCCG
sPEDF^146A/147A/149A^	TCGTCTTTGAGGCGGCGCTGGCCATAAAATCCAG
asPEDF^146A/147A/149A^	CTGGATTTTATGGCCAGCGCCGCCTCAAAGACGA
sPEDF-F^63F/66F/67Q/69Q/70A^	AACTTCGGCTTTGACCTGTTCCAGGTGCAAGCCATGAGCCCC
asPEDF^63F/66F/67Q/69Q/70A^	GGGGCTCATGGCTTGCACCTGGAACAGGTCAAACCGAAGTT
sPEDF^48A/53A^	CCTTTCTTCGCAGTCCCCGTGAACGCGCTGGCAGCG
asPEDF^48A/53A^	CGCTGCCAGCGCGTTCACGGGGACTGCGAAGAAAGG
sPEDF^67Q/69Q^	GGCTATGACCTGTACCAGGTGCAATCCAGCATGAGCCCC
asPEDF^67Q/69Q^	GGGGCTCATGCTGGATTGCACCTGGTACAGGTCATAGCC
sTRNSR2	CCGGATCCAACATTTGCTCTGTCTGC
asTRNSR2	CGGAATTCCTATCGAAACAACCTGGTG

To detect expression of transportin in cell lines RT-PCR amplification of a 1197 bp region of the gene coding sequence was performed using the primers sTRNSR2 and asTRNSR2.

Site directed mutagenesis was carried out according to the QuikChange® site-directed mutagenesis kit (Stratagene) using the relevant primers shown in Table 1. Sequencing of mutants (GATC Biotech) was performed to verify expected sequence changes.

### Yeast-two-hybrid library screen

Residues 41–121 of PEDF fused to the DNA binding domain of LexA were used as a bait to screen a human fetal brain cDNA library (Origene) for potential interactors using the 2-hybrid system described [38]. The bait vector pEG202-PEDF^41–121^ and the library target vector pJG4-5 were sequentially transformed [39] into the yeast strain EGY48 and grown on media lacking leucine to select for potential interactors. Approximately 1.5×10^6^ clones representing approximately 42% of the library were screened against the PEDF bait construct. Positive transformants were analyzed by PCR amplification of the target inserts using primers BCO1 and BCO2 to the vector arms of pJG4-5. Clones that produced unique inserts were transformed into KC8 *E.coli* and sequenced. The sequences obtained were identified using BLASTN.

### HEK 293T transfection

HEK293T cells were grown to 50% confluency and transfected using a modified calcium phosphate method [40]. For stable transfections, cells were kept in selection medium containing 500 µg/ml G418 for two weeks and fluorescent cells were subsequently enriched by fluorescence-activated cell sorting.

For Transportin SR2 knockdown cells were grown to 50% confluency and transfected using oligofectamine (Life Technologies) with a synthetic siRNA targetting nucleotides 605–624, or a control mismatch targetting the same nucleotides, harbouring four mutations as described [27].

### Recombinant protein expression and purification

Recombinant human PEDF was recovered and purified from conditioned medium of transiently transfected HEK293T cells.

Cells were grown to 50% confluency and transiently transfected as described above with pCMV-PEDF or pCMV-PEDF^R67Q-R69Q^ expression vectors. Following an overnight incubation at 37°C, the media was replaced twice with serum free DMEM supplemented with 2 mM glutamine, 100 U/ml penicillin and 100 µg/ml streptomycin. After 24 hr incubation the conditioned medium was collected, pooled and PEDF was purified on Heparin-agarose (Sigma). Conditioned medium was loaded onto a pre-packed heparin-agarose column, washed with binding buffer (20 mM Tris-HCl pH 7.4) and recombinant PEDF or PEDF^R67Q-R69Q^ were eluted with binding buffer containing 100 mM NaCl.

For expression and purification of GST-Transportin-SR2, pGEX-TRN-SR2 (kindly donated by Dr W.Y. Tarn, Academia Sinica, Taipei) was transformed into BL21 *E.coli* strain and grown at 37°C overnight. After induction with IPTG (1 mM) for 6 hr at 22°C bacteria were centrifuged at 10,000 g for 15 min at 4°C and lysed with bugbuster lysis buffer (Novagen) for 15 min. Lysates were centrifuged at 24,000 g for 15 min at 4°C, filtered through a 20 µm syringe filter and applied to a pre-packed glutathione agarose column. The column was washed with PBS and GST-TRN-SR2 eluted with PBS containing 5 mM free glutathione. Eluates were concentrated and subjected to buffer exchange with PBS in 10000 kDa cutoff centrifugal filter units (Millipore). Purified proteins were verified by immunoblotting and protein concentration was quantified using the Pierce (Rockford) BCA Micro Protein Assay.

### SDS-PAGE and Immunoblotting

For immunoblotting of cell extracts, cells were rinsed with ice-cold PBS, resuspended in lysis buffer (62.5 mM Tris-HCl pH 6.8, 2% SDS, 10% glycerol, 12.5 mM EDTA) and denatured at 95°C for 15 min. Protein samples (20–50 µg) were separated on 10% SDS-PAGE and blotted to nitrocellulose membranes (Protean BA 85; Schleicher & Schuell). The membranes were blocked in TBS supplemented with 5% non-fat milk (Sigma Aldrich), 0.1% Tween-20 for 1 hr at room temperature and incubated overnight with the primary antibody. The following primary antibodies were used: a rabbit polyclonal actin (Santa Cruz), a rabbit polyclonal GFP (Abcam, Cambridge, UK), a mouse monoclonal GST (Sigma Aldrich), a mouse monoclonal HA (Babco), a rabbit polyclonal Lamin (Santa Cruz), a rabbit polyclonal Lex A (Invitrogen), a mouse monoclonal PEDF (Millipore), a mouse monoclonal Transportin-SR2 TNPO3 (Abcam). Horseradish peroxidase-conjugated secondary antibodies (Cell Signalling) were incubated for 1 hr at room temperature and detected using SuperSignal West Pico Chemiluminescent Substrate (Pierce).

### Co-Immunoprecipitation and GST Pulldown

For pull down experiments GST or GST-TRN-SR2 were absorbed on glutathione-agarose for 1 hr followed by addition of purified recombinant PEDF or PEDF^R67Q-R69Q^ for 4 hr at 4°C in binding buffer (50 mM Tris-HCl pH 7.5, 150 mM NaCl, 1 mM EDTA, 1 mM DTT, 5% glycerol). Samples were washed with ice cold binding buffer containing 1% CHAPS (Sigma), centrifuged for 30 sec. at 13,000 g and processed by SDS page and immunoblotting.

For immunoprecipitation PEDF or PEDF^R67Q-R69Q^ were incubated with GST or GST-TRN-SR2 for 4 h and monoclonal PEDF antibody pre-absorbed on Protein-A agarose for 1 h at 4°C in binding buffer (50 mM Tris-HCl pH 7.5, 150 mM NaCl, 1 mM EDTA, 1 mM DTT, 5% glycerol). Samples were washed with ice cold binding buffer containing 1% CHAPS (Sigma), centrifuged for 30 sec. at 13,000 g and processed by SDS page and immunoblotting.

### Subcellular fractionation

HEK293T cells were transiently transfected with the pEGFP-C1-PEDF and fractionated into cytoplasmic and nuclear fractions as described [41]. Briefly, cells were collected by centrifugation at 6000 rpm at 4°C for 2 min, and swelled in hypotonic buffer (10 mM Hepes pH 7.9, 10 mM KCL, 0.1 mM EDTA) containing complete mini protease inhibitor cocktail (Roche) for 15 min on ice. Lysis was achieved by adding 0.63% v/v Nonidet-P40 and vortex mixing for 10 sec. Nuclei were separated from cytoplasm by centrifugation at 14,000 rpm for 15 sec, and subsequently incubated in 500 µl lysis buffer (20 mM Hepes, 0.4 M NaCl, 1 mM EDTA, 10% glycerol, supplemented with protease inhibitors) for 15 min. on ice. The supernatant (i.e. nuclear fraction) was cleared by further centrifugation at 13,000 rpm for 15 min. at 4°C. For subcellular fractionation of mouse liver tissue, the Sigma Cell Lytic NuClear™ Extraction kit was used according to manufacturers instructions.

### Immunocytochemistry

3×10^4^ cells were seeded on 13 mm coverslips in 24 well plates. Cells were fixed with 4% paraformaldyde 4% sucrose in PBS, permeabilized with PBS 0.1% Triton X-100 for 3 min. on ice and blocked with PBS 0.3% Triton x-100 5% donkey serum for 2 hours. Cells were incubated with 1 mM DAPI and mounted on glass slides.

### Confocal microscopy and subcellular localisation studies

Glass slides were mounted on the microscope stage and images were recorded through a 63× objective using a Zeiss LSM-5-Meta confocal microscope equipped with a 20 mW UV, a 20 mW 488 nm, a 20 mW 543 nm and a 20 mW 633 nm laser.

DAPI was acquired through a 385–470 nm band pass filter using 5% of the UV laser intensity; GFP was acquired through a 505–530 nm band pass filter using 5% of the 488 nm laser intensity. Single plane images were exported and analyzed with Image J. In order to assess nuclear to cytoplasmic fluorescence ratio a mask in the DAPI fluorescence image was used to identify the nucleus and a mask in the GFP fluorescence image was used to identify the whole cell. GFP nuclear fluorescence was collected for all the cells analysed and subtracted to the total cell fluorescence to generate cytoplasmic fluorescence. For each single cell analysed the nuclear to cytoplasmic fluorescence ratio was calculated by dividing the nuclear GFP fluorescence by the cytoplasmic GFP fluorescence. Data are shown as mean +/− SD.

### Flow Cytometry

Cells were removed from the tissue culture plates with trypsin, washed with PBS twice and incubated with PBS or digitonin (2 µg/ml) at 4°C for 10 min. Samples were acquired on a Cyan flow cytometer (Beckman Coulter) equipped with a 20 mW 405 nm, 20 mW 488 nm and 20 mW 633 nm lasers. GFP fluorescence was excited with the 488 nm laser and fluorescence emission was acquired through a 520+/−20 nm band pass filter. Data are reported as median of the fluorescence peak of 3 experiments performed in triplicate.

### Statistics

Data are given as means ± SD or SEM. For statistical comparison, t-test or one-way ANOVA followed by the Tukey test were employed using SPSS software (SPSS GmbH Software, Munich, Germany). P-values smaller than 0.05 were considered to be statistically significant.
